# Local application of doxorubicin- loaded Iron oxid nanoparticles and the vascular disrupting agent via the hepatic artery: chemoembolization–photothermal ablation treatment of hepatocellular carcinoma in rats

**DOI:** 10.1186/s40644-019-0257-x

**Published:** 2019-11-04

**Authors:** Hongjun Yuan, Xin Li, Jing Tang, Min Zhou, Fengyong Liu

**Affiliations:** 1 Department of Interventional Radiology, The First Medical Center of PLA General Hospital, Beijing, 100853 China; 2 Institute of Translational Medicine, Zhejiang University, Hangzhou, 310009 China

**Keywords:** Iron oxide nanocomposites, Chemoembolization, Photothermal ablation, Hepatocellular carcinoma, CA4P

## Abstract

**Objectives:**

This study investigates the effectiveness of local application of doxorubicin(Dox)-loaded, polydopamine (PDA)- coated single crystal hematite (α- Fe_2_O_3_) nanocubes (Fe_2_O_3_-PDA-Dox) and combretastatin A-4 phosphate disodium(CA4P)in treating hepatocellular carcinoma (HCC) in rats.

**Methods:**

The magnetic characteristics and photothermal effects of the nanoparticles were determined in vitro*.* Tumor-bearing Sprague–Dawley rats were divided into 3 groups of 8 according to treatment: controls, transarterial chemoembolization–photothermal ablation (pTACE) (Lipidol+Fe_2_O_3_-PDA-Dox + NIR), and CA4P + pTACE (CA4P+ Lipidol+Fe_2_O_3_-PDA-Dox + NIR). Drugs were administered through the hepatic artery, and the tumors exposed to 808-nm near-infrared radiation. The Fe content of tumors was assessed using neutron activation analysis. Treatment effectiveness was assessed using heating curves, magnetic resonance imaging, pathology results, and immunohistochemical analysis.

**Results:**

The mean tumor Fe content was greater in rats treated with CA4P + pTACE (1 h, 23.72 ± 12.45 μg/g; 24 h, 14.61 ± 8.23 μg/g) than in those treated with pTACE alone (1 h, 5.66 ± 4.29 μg/g; 24 h, 2.76 ± 1.33 μg/g). The tumor T2 imaging signal was lower in rats treated with CA4P + pTACE. Following laser irradiation, the tumor temperature increased, with higher temperatures reached in the CA4P + pTACE group (62 °C vs 55 °C). Tumor cells exhibited necrosis, apoptosis, and proliferation inhibition, with greater effects in the CA4P + pTACE group. Transient liver and kidney toxicity were observed on day 3, with more severe effects after CA4P + pTACE.

**Conclusions:**

Fe_2_O_3_-PDA-Dox nanoparticles are effective for TACE–PTA. Pretreatment with CA4P increases nanoparticle uptake by tumors, increasing the treatment effectiveness without increasing hepatorenal toxicity.

## Introduction

While transcatheter arterial chemoembolization (TACE) is an important method for treating intermediate and advanced stage HCC [1], tumor necrosis after TACE treatment is not complete, and recanalization of remnant tumor vessels is currently a major clinical concern [1–4]. Recent developments in nanotechnology have attracted great interest in the use of nano-scale photothermal materials in conjunction with chemotherapeutic drugs for the treatment of tumors [5]. Fe_2_O_3_ has been approved by the US Food and Drug Administration for use as a metal nanomaterial [6] for such purposes. This paramagnetic material can significantly shorten the magnetic resonance imaging (MRI) lateral relaxation time, has a characteristic low signal in T2 imaging, can be used as a T2 negative-contrast agent [7], and allows for monitoring of nanoparticle distribution within the body. Polydopamine (PDA), a biopolymer commonly used for drug delivery, can be linked to the chemotherapeutic agent doxorubicin (Dox). In the acidic microenvironment of a tumor, the pH sensitivity of PDA results in its release of Dox to the target site [8]. PDA exerts further tumoricidal effects through photothermal ablation (PTA) in response to near-infrared radiation (NIR) at a wavelength of 808 nm [9–11]. We designed an Fe_2_O_3_-PDA-Dox nanoparticle system capable of releasing Dox under low-pH conditions and facilitating T2 MRI imaging.

This study investigates the effects of the Fe_2_O_3_-PDA-Dox nanoparticle system in combination with combretastatin A4-phosphate (CA4P), a vascular disrupting agent, on HCC in rats. We included CA4P in the treatment because it specifically blocks and destroys vessels supplying blood to tumors, inhibits the formation of new blood vessels, and increases the permeability of blood vessels and cell membranes [12, 13]. We believed that this approach to treatment could maximize the accumulation of chemotherapeutic drugs in the tumor locale, reduce toxic side effects, and achieve synergistic PTA and chemoembolization in the treatment of HCC. After injecting nanoparticles through the hepatic artery, treatment effectiveness with and without pretreatment with CA4P was assessed using Fe distribution analysis, heating curves, imaging, pathology results, and immunohistochemical analysis.

## Materials and methods

### In vitro experiments

#### Fe_2_O_3_-PDA synthesis and Dox loading

The microwave hydrothermal (MH) method was used to synthesize ferric oxide nanocrystals [14]. Fe_2_O_3_ nanoparticles (100 mg) were dissolved in 100 mL Tris-HCl buffer solution (pH 8.5, 10 mmol/L), to which was added dopamine hydrochloride (25 mg). The solution was mixed in the dark for 8 h and then centrifuged to obtain Fe_2_O_3_ nanoparticles modified with PDA (Fe_2_O_3_-PDA). Fe_2_O_3_-PDA nanocrystals (100 mg) were added to an aqueous solution of Dox (1 mg/mL, 40 mL) at room temperature. The solution was mixed overnight in the dark and then centrifuged to collect the Fe_2_O_3_-PDA nanoparticles carrying dox.

#### Release of Dox by Fe_2_O_3_-PDA-Dox nanoparticles

Fe_2_O_3_-PDA-Dox (25 mg) was added to 10 mL phosphate buffer solution (PBS) at pH of either 7.4 or 6.5, and the solutions were agitated at 37 °C until homogeneous. Ultraviolet–visible spectrophotometry was employed at wavelengths under 478 nm to calculate the release of Dox molecules at the different pH values. Percentage release was calculated as (cumulative amount of Dox released each time)/(total amount of Dox carried by the Fe_2_O_3_-PDA nanoparticles) * 100%.

#### In vitro magnetic properties of Fe_2_O_3_-PDA- Dox nanoparticles

Nanoparticle solutions of 0, 6.25 μg/mL, 12.5 μg/mL, 25 μg/mL, 50 μg/mL, 100 μg/mL, and 200 μg/mL were added to 1.5 Eppendorf tubes, and T2WI images were obtained using a 3.0 T MRI scanner (scanning parameters: use of a wrist coil; slice thickness, 2.0 mm; number of slices, 16).

#### Photothermal effect of in vitro Fe_2_O_3_-PDA-Dox nanoparticles

A fiber optic-coupled near-infrared laser (MDL-H-808-5 W) was used in vitro as a laser light source to test the photothermal response of Fe_2_O_3_-PDA-Dox nanoparticles at 0, 5 μg/mL, 10 μg/mL, and 20 μg/mL. After near-infrared irradiation with a wavelength of 808 nm and a power density of 2.0 W/cm^2^ for 5 min at room temperature, a forward-looking infrared (FLIR) camera was used to monitor temperature changes.

### In vivo animal experiments

#### Establishment of a rat model of hepatoma in situ

Mca-rh7777 rat hepatoma cells from the American Type Culture Collection Center (CRL-1601) were provided by Shanghai Rongbai Biological Technology Co., Ltd. After revival and culture, 4 × 10^6^ cells were suspended in PBS and slowly injected into the rat liver using a 1-mL syringe. After this procedure, the rats were given antibiotics and dexamethasone intramuscularly for 3 days. Tumors formed within one week. The tumor-bearing rats were randomly divided into three groups according to treatment: control, pTACE (Lipidol + Fe_2_O_3_-PDA-Dox + NIR), and CA4P + pTACE (CA4P + Lipidol + Fe_2_O_3_-PDA-Dox + NIR), with 8 rats in each group.

#### Hepatic artery chemoembolization (retrograde intubation of the hepatic artery via the gastroduodenal artery)

A midline incision was made, and the stomach was exposed and turned over to locate the gastroduodenal artery. The hepatic artery was freed along the gastroduodenal artery. The gastroduodenal artery was punctured using a 24G venous cannula, and a 0.014-in. micro-guide wire made in the laboratory was inserted into the sheath of the cannula. The guide wire was selectively pushed into the hepatic artery proper, and the cannula sheath was pushed into the hepatic artery along the guide wire. The guide wire was then pulled out, and a micro catheter was superselectively inserted into the hepatic artery. Each group was administered a different drug combination, with the pTACE group given 1 mL iodinate oil mixed with 1 mL of Fe_2_O_3_-PDA-Dox (20 μg/mL), with NIR of the tumor location performed 3 h after injection. The CA4P + pTACE group first was given 0.5 mL 0.1 mg/mL CA4P, followed by infusion with 1 mL iodinate oil mixed with 1 mL of Fe_2_O_3_-PDA-Dox (20 μg/mL), with NIR irradiation performed 3 h after injection.

#### Photothermal ablation

An MDL-H-808-5 W near-infrared laser (Changchun New Industries) was used for irradiation using NIR with a wavelength of 808 nm. After selecting a fiber optic cable, irradiation was performed at a distance of 40 cm and output power of 5 W using a diaphragm with a diameter of 1.2 cm aimed at the tumor area. The optical power density in the tumor area was approximately 4.4 W/cm^2^, and the irradiation time was 5 min. Following irradiation, the rat abdomens were closed and sutured. To prevent infection, 300,000 units of postoperative penicillin sodium was administered to each rat for 3 days.

#### Neutron activation analysis, MRI imaging, and assessment of hepatorenal function

Fe content of tumor tissue and peripheral normal liver tissue was determined at 1 h and 24 h after injection using neutron activation analysis after injection. MRI examination of rats in all three groups was performed before surgery and at 3, 7, and 10 days after injection using the examination method described. Coronal and axial T2 fat suppression (FS) images were acquired before and after treatment. The longest diameter and transverse diameter of each tumor were measured. Blood (1 mL) for biochemical analysis was drawn from the rat tail vein before surgery and at 3, 7, and 14 days after injection, and the concentrations of alanine transaminase (ALT), aspartate transaminase (AST), total bilirubin (TBIL), and creatinine (CREA) were compared.

### Statistical analysis

All quantitative data are expressed as mean ± standard deviation (SD). Comparative analysis was carried out using SPSS 23.0. Groups were compared using the t-test, and multi-group comparisons employed one-way ANOVA. The testing standard was α = 0.05, and P < 0.05 indicated a statistically significant difference. Graphpad Prism 7.0 software was employed for charting.

## Results

### Loading of Dox onto Fe_2_O_3_-PDA nanoparticles and pH-triggered release

Ultraviolet–visible spectra (Fig. S1a) reveal that Fe_2_O_3_-PDA-Dox and Dox have similar characteristic peaks at approximately 478 nm, indicating that the Fe_2_O_3_-PDA nanoparticles successfully carried the Dox molecules. The in vitro drug-release curves of Fe_2_O_3_-PDA-Dox (Fig. S1b) indicate that in a PBS solution of neutral pH (7.4), the Fe_2_O_3_-PDA-Dox nanoparticles released roughly 2.0% of their Dox within 10 h and 7.5% of their Dox within 28 h; at a pH of 6.5, simulating the acidic environment of tumor tissue, 29 and 32% of the Dox was released at 10 h and 28 h, respectively.

### Magnetic properties of Fe_2_O_3_-PDA-Doxnanoparticles

Concentration-dependent (0–200 μg/mL) signal attenuation (Fig. S2) was seen in T2WI images, indicating the feasibility of using MRI to monitor the in vivo treatment process and verifying the use of Fe_2_O_3_-PDA-Dox nanoparticles as a negative MRI contrast agent.

### Photothermal response of Fe_2_O_3_-PDA-Dox nanoparticles

The nanoparticle temperature increased steadily with increasing in vitro irradiation time (Fig. S3a). The heating curve (Fig. S3b) shows that the temperature gradually increased as the Fe_2_O_3_-PDA-Dox concentration increased from 0 to 20 μg/material. After irradiation for 300 s., the temperature of pure water increased only 3.5 °C (room temperature, 24.5 °C) but increased roughly 35 °C at a nanoparticle concentration of 20 μg/mL, which was the greatest temperature increase.

#### In vivo MRI monitoring of Fe_2_O_3_-PDA-Dox distribution

**Fig. 1:**
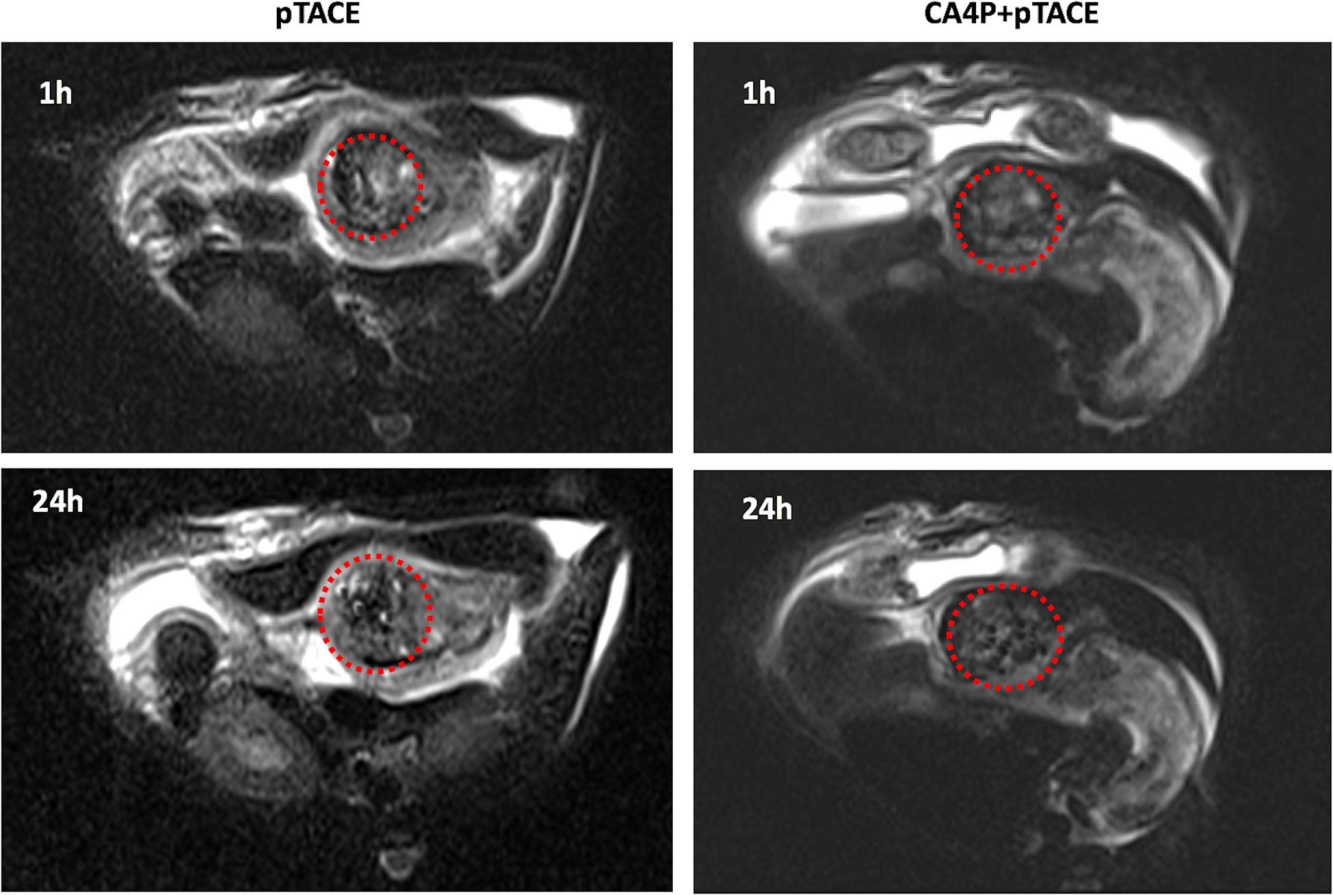
T2WI imaging of rats in the pTACE and CA4P + pTACE groups at 1 h and 24 h after injection of Fe_2_O_3_-PDA-Dox. The rat hepatoma tissue is located within the red circle

T2 MRI images were acquired at 1 h and 24 h after injection following the injection of Fe_2_O_3_-PDA-Dox nanoparticles (after CA4P injection in some groups) via the hepatic artery. The in vivo Fe_2_O_3_-PDA-Dox content of the tumor was assessed (Fig. 1). T2 image signals from the tumor were even lower at 24 h than at 1 h in both groups, indicating that most of the Fe_2_O_3_-PDA-Dox had entered the tumor tissue. The T2 signal in the CA4P + pTACE group was lower than that of the pTACE group at both 1 h and 24 h after injection, which indicates that the relaxation time of the tumor tissue was shorter in the CA4P + pTACE group, and the tumor tissue in this group contained more Fe_2_O_3_-PDA-Dox particles. This observation indicates that CA4P promotes the uptake of Fe_2_O_3_-PDA-Dox by tumor tissue.

#### Neutron activation analysis of tissue Fe content

**Fig. 2:**
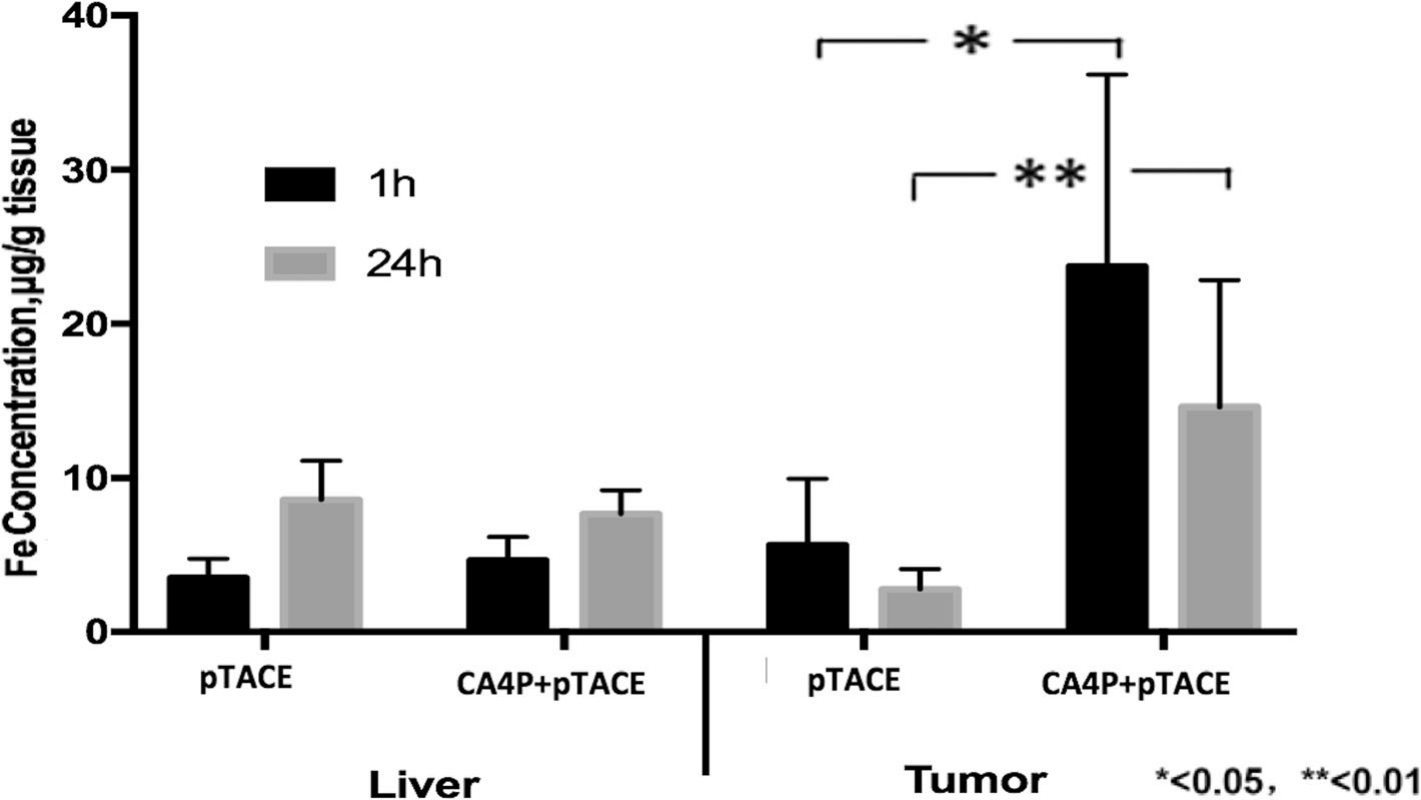
Fe content of tumor tissue and liver tissue in rats treated with CA4P + pTACE and pTACE alone at 1 h and 24 h following injection of Fe_2_O_3_-PDA-Dox

The mean Fe content of tumor tissue in the CA4P + pTACE group at 1 h and 24 h after injection was greater than that of normal liver tissue (23.72 ± 12.45 μg/g and 14.61 ± 8.23 μg/g; normal tissue, 4.66 ± 1.52 μg/g and 7.67 ± 1.35 μg/g, respectively). The mean Fe content of tumor tissue in the pTACE group differed much less from that of normal tissue at 1 h and 24 h after injection (5.66 ± 4.29 μg/g and 2.76 ± 1.33 μg/g; normal liver, 3.53 ± 1.23 μg/g and 8.58 ± 2.64 μg/g, respectively) (Fig. 2). This result indicates that while there was no significant difference in liver uptake of Fe_2_O_3_-PDA-Dox between these two groups, Fe_2_O_3_-PDA-Dox uptake was significantly higher in the in tumors treated with CA4P + pTACE than in those treated with pTACE alone.

#### Photothermal ablation

**Fig. 3:**
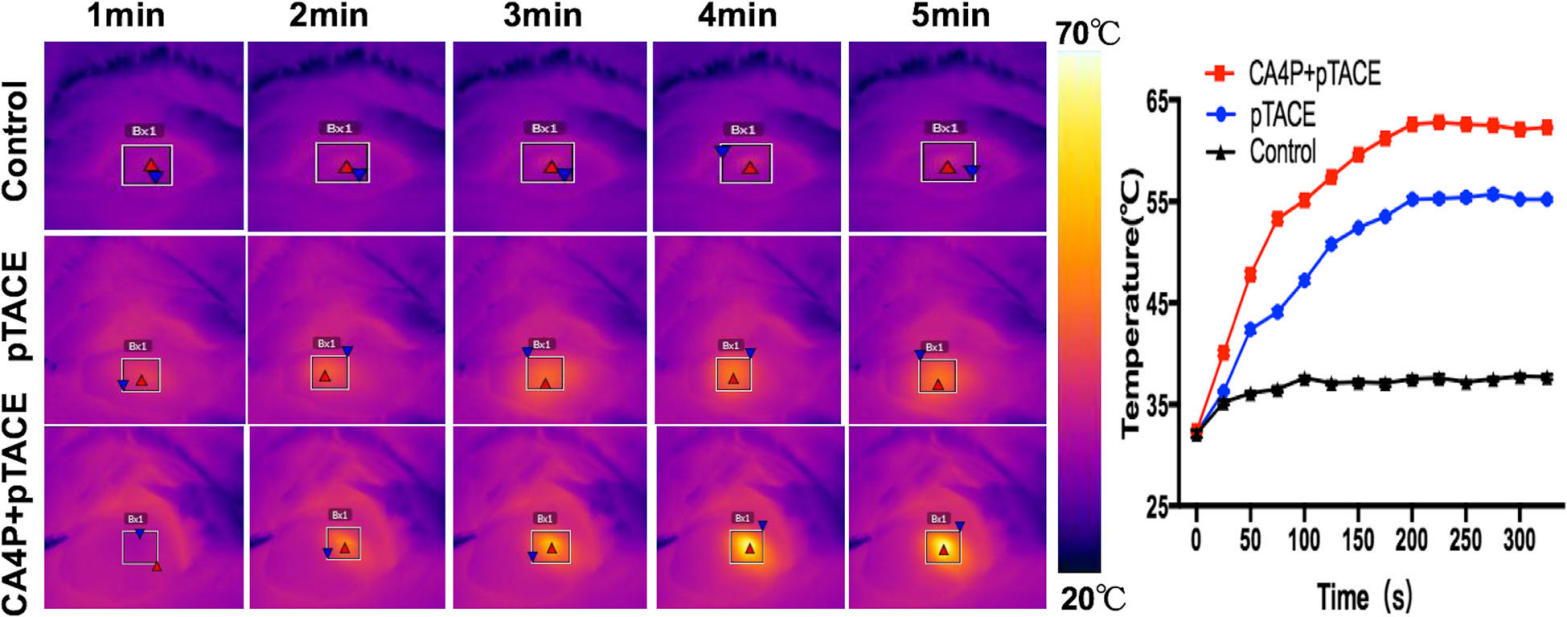
In vivo PTA of tumor areas in rats and heating curves

The temperature of the tumor area rose significantly in both groups following the injection of Fe_2_O_3_-PDA-Dox nanoparticles. In the pTACE group, the temperature increased to 42 °C and 55 °C at 1 and 5 min after near infrared laser irradiation, respectively. Greater temperature increases were observed in the CA4P + pTACE group, to 47 °C and 62 °C at 1 and 5 min after irradiation, respectively. The control group displayed a temperature increase of less than 5 °C following irradiation. The resulting heating curve (Fig. 3) indicates that co-injection of CA4P promotes the uptake of Fe_2_O_3_-PDA nanoparticles by tumor tissue, with the temperature in the tumor area rising to over 60 °C within 5 min after irradiation. This temperature can effectively kill tumor cells.

#### MRI assessment of tumor treatment effectiveness

**Fig. 4:**
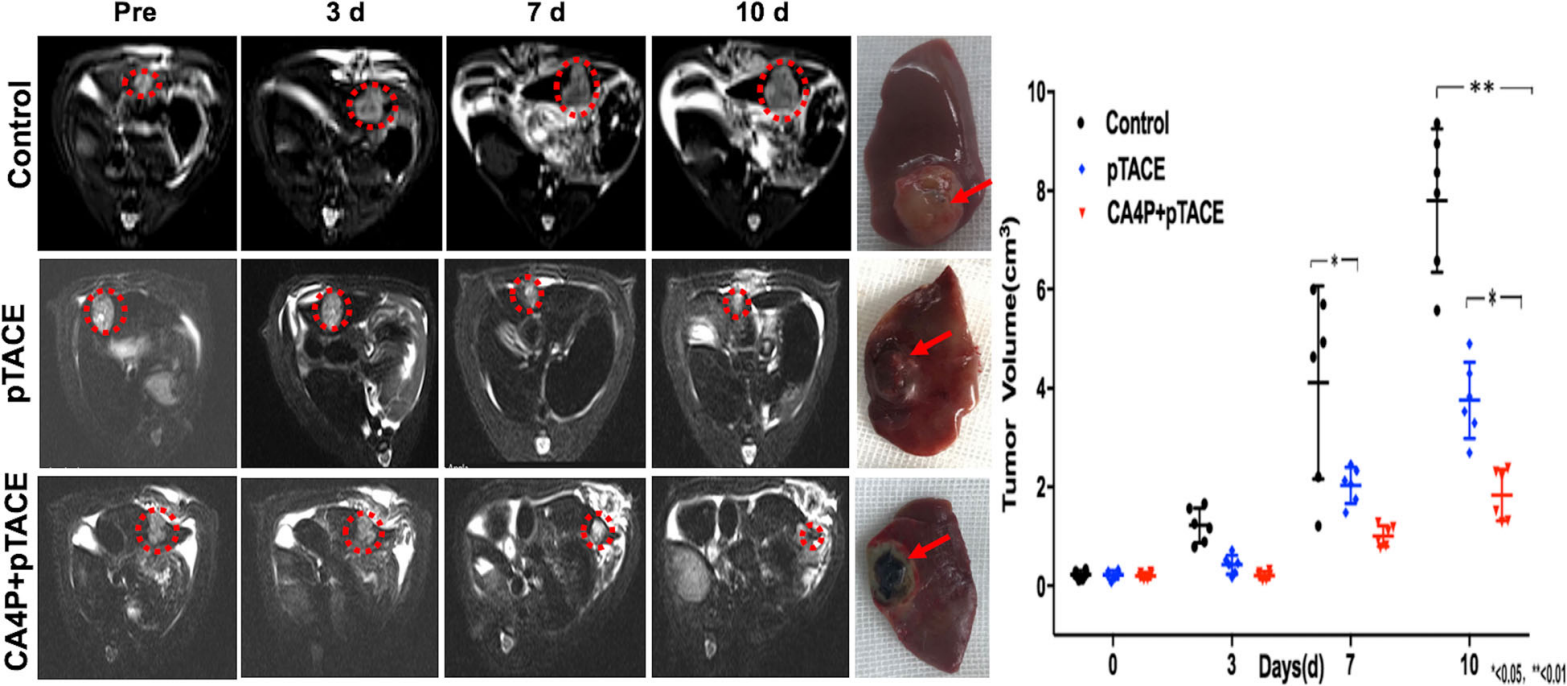
In vivo MRI dynamic monitoring of tumor growth and volume changes in an in-situ rat hepatoma model following injection of Fe_2_O_3_-PDA-Dox nanoparticles via the hepatic artery

MRI examination of liver tissue in the control, pTACE, and CA4P + pTACE groups was performed before and at 3 d, 7 d, and 10 d after injection to dynamically assess tumor necrosis and changes in tumor volume (Fig. 4). On day 10, the tumor volume was 7.53 ± 1.72 cm^3^ (control), 2.14 ± 0.24 cm^3^ (pTACE), and 0.43 ± 0.11 cm^3^ (CA4P + pTACE). The tumors in the control group grew steadily, and T2WI images displayed a slightly elevated signal. By comparison, the tumor volume was significantly lower in the pTACE and the CA4P + pTACE groups, and T2WI images displayed a slightly reduced signal. While the difference in volume change between the pTACE group and control group at 10 d was not significant, that between the CA4P + pTACE group and controls differed significantly.

#### Pathology and immunohistochemistry results

**Fig. 5:**
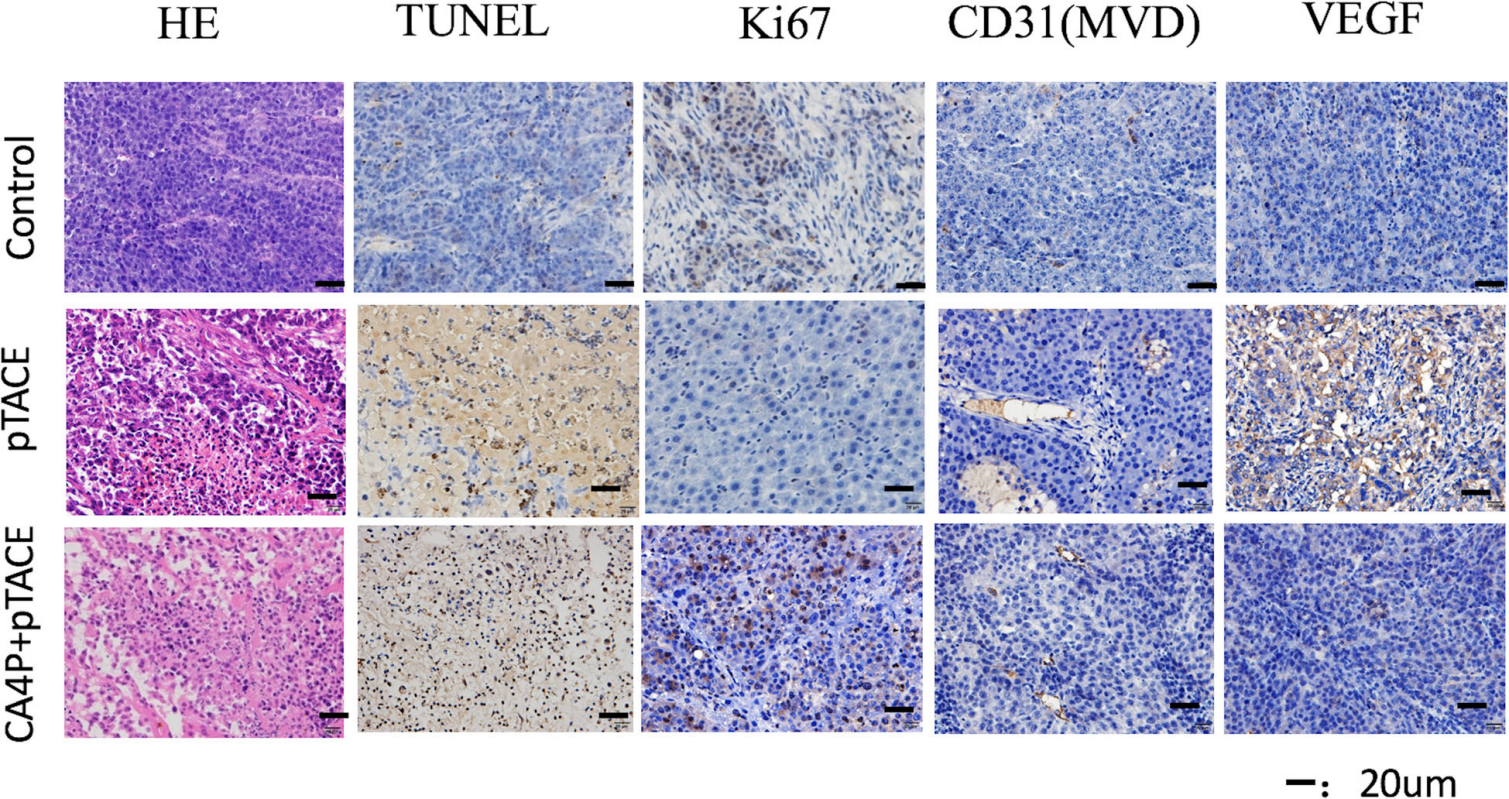
H & E and TUNEL staining of Caspase3, Ki-67, CD31, and VEGF in tumor tissue sections from rats in each group

H & E staining revealed that rats in the pTACE and CA4P + pTACE groups had small numbers of malformed, disarranged tumor cells, some of which appeared in the form of broad stripes. Necrosis was visible in most tissues, and infiltration by small numbers of inflammatory cells, as well as moderate amounts of fibrous tissue, were observed. Microscopic analysis of TUNEL-stained tissues revealed apoptosis in 26.27 ± 5.65% of the tumor cells in the pTACE group and 31.58 ± 6.82% of the tumor cells in the CA4P + pTACE group. Differences in the expression of tumor markers between the pTACE and CA4P + pTACE groups as indicated by immunohistochemical staining were as follows: Ki67, 0.75 ± 0.67% vs 0.42 ± 0.42%; CD31 (MVD),10.6 ± 4.16% vs 8.40 ± 4.01%; VEGF, 148.63 ± 42.48% vs 129.76 ± 36.03%, respectively. The low level of Ki-67 expression indicates significant tumor cell necrosis, apoptosis, and inhibition of proliferation in both groups after treatment. Angiogenesis was lower in tumors of animals treated with CA4P + pTACE than in those treated with pTACE alone, and the expression of CD31 and VEGF was qualitatively reduced accordingly (Fig. 5).

#### Safety: Hepatorenal toxicity

**Fig. 6:**
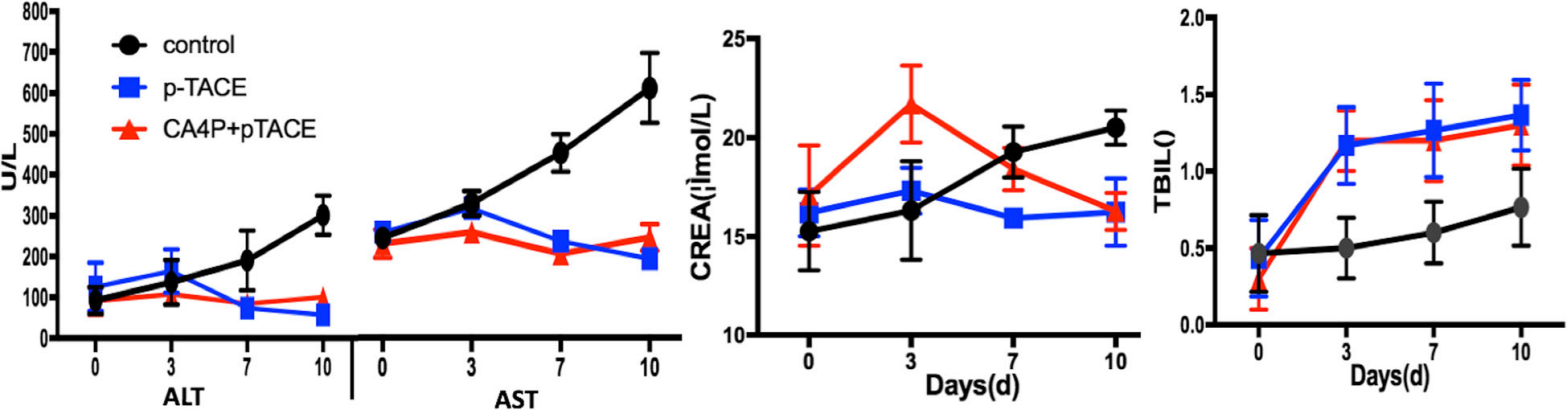
Dynamic assessment of hepatorenal toxicity (ALT, AST, CREA, and TBIL) in the two groups during treatment

Transient liver and kidney toxicity were observed in both groups on day 3. The CA4P + pTACE group sustained more damage than did the pTACE group. The toxic effects gradually decreased on days 7 and 10. By day 10, the AST, ALT, and CREA levels were normal. Indicative of bile duct damage, TBIL levels increased slightly in both groups (Fig. 6), with no significant difference between the groups.

## Discussion

Minimally invasive, interventional therapy is currently recognized as an important means of HCC [15, 16]. New forms of interventional therapy have emerged with technological advances. Improvements in drug-carrying chemoembolization materials and noninvasive physical ablation methods would be of great clinical benefit [17, 18]. Of the many nano-biomaterials, multifunctional nanomaterials used in multimode imaging and chemoembolization–PTA (TACE-PTA) with imaging guidance [19] have attracted widespread attention, and the potential of multifunctional iron-based nanomaterials in clinical applications is particularly promising. We therefore designed and investigated the function of nanoparticles carrying Fe_2_O_3_-PDA-Dox for treating HCC. Our results show that treatment with CA4P improves the tumor uptake of nanoparticles carrying Fe_2_O_3_-PDA-Dox, increasing the effect of TACE and PTA to provide increased anti-tumor activity.

### MRI, optical characteristics, and pH-triggered release of nanoparticles carrying Fe_2_O_3_-PDA-Dox

Because the iron in Fe_2_O_3_-PDA-Dox nanoparticles possesses superparamagnetism at room temperature, Fe_2_O_3_-PDA-Dox carrying nanoparticles will generate a strong local magnetic field in the presence of an external magnetic field, which strongly influences the relaxation of hydrogen protons in the water molecules around the particles. This effect shortens the T2 time of the hydrogen protons and causes T2 weighted images to appear dark [7, 20]. We employed an in vitro experiment to further verify the ability of nanoparticles carrying Fe_2_O_3_-PDA to serve as negative contrast agents in MRI. Our results show that the T2 signal strength decreases with increasing concentrations of Fe_2_O_3_-PDA-Dox. PDA nanoparticles with a photothermal conversion function were first reported in 2013 by Liu et al. [21]. PDA possesses photothermal properties and offers relatively high photothermal conversion efficiency. As a consequence, we chose to use PDA as the primary photothermal material in the nanoparticles. Fe_2_O_3_-PDA-Dox nanoparticles have high absorbance in the near infrared zone and displays excellent in vitro heating when irradiated by NIR with a wavelength of 808 nm [9–11]. Monitoring of the solution temperature with a thermal imager verified the in vitro heating effect of Fe_2_O_3_-PDA-Dox nanoparticles following NIR, and in vivo results showed that the temperature of tumor tissue rose to 63.4 °C after irradiation for 5 min, a temperature sufficient to kill tumor tissue [22, 23]. While the covalent bonds of Fe_2_O_3_-PDA are highly effective at carrying dox [24], the pH-sensitivity of PDA resulted in the release of Dox into simulated tumor tissue at pH 6.5, with 29 and 32% of the Dox content released at 10 and 28 h, respectively. Since the amount of drug released at pH 6.5 was far greater than at pH 7.0, this agent can be used for selective drug release during treatment. In summary, our results indicate that nanoparticles carrying Fe_2_O_3_-PDA-Dox constitute a nano-scale drug-carrying system with the attributes of pH-triggered drug release, capacity for MRI monitoring, photothermal responsiveness, and the ability to simultaneously achieve TACE and photo-thermal ablation effects.

### Study on the use of CA4P in conjunction with Fe_2_O_3_-PDA-Dox nanoparticles to treat HCC

The majority of HCC tumors are well supplied with blood from a variety of arteries [25]. As a consequence, necrosis is not complete following TACE treatment of some tumors [4, 25]. The use of angiogenesis inhibitors targeting new blood vessels to increase the effectiveness of TACE has become an area of research interest [26, 27]. Binding of the active form of CA4P to the microtubule system of the vascular endothelium blocks angiogenesis, resulting in anti-tumor effects [28]. Głowacka et al. [29] verified that CA4P increases the permeability of endothelial cells by blocking the VE-cadherin/β-catenin/Akt pathway, while simultaneously inhibiting the migration of endothelial cells and the formation of capillaries. This effect rapidly leads to vascular disruption and necrosis of tumor tissue. In view of the local nature of TACE and the characteristics of CA4P and Fe_2_O_3_-PDA-Dox–carrying nanoparticles, we believed that CA4P could increase the permeability of tumor blood vessels, thereby increasing the uptake of Fe_2_O_3_-PDA-Dox nanoparticles by tumor tissue, resulting in increased effectiveness of TACE–photo-thermal ablation nanoparticles against tumors. A study by Wang et al. [30] suggests that a vascular disrupting agent is effective if administered 1–4 h before chemotherapy. Tozer et al. [31] reported that blood flow to tumors is reduced by 95% only 1 h after injection of CA4P, showing that extensive destruction of tumor capillaries occurs within 1 h of its administration. Using MRI monitoring of Fe_2_O_3_-PDA-Dox distribution, we observed that a decrease in the tumor T2 signal during the first 24 h after injection in the CA4P + pTACE group. Fe_2_O_3_-PDA-Dox particles accounted for most of this effect, and they became increasingly evenly distributed with time. Neutron activation analysis to measure Fe content revealed that while the uptake of Fe_2_O_3_-PDA by normal liver tissue did not differ significantly at 1 h and 24 h, the uptake of Fe_2_O_3_-PDA-Dox at 1 h and 24 h in the CA4P + pTACE group was significantly greater than in the pTACE group. These results verified that pre-injection of CA4P via the hepatic artery before TACE increases the uptake of nanoparticles by tumor tissue. Heating curves show that the temperature increase in tumors treated with TACE was greater in rats pre-treated with CA4P, indicating that CA4P increases the effectiveness of PTA. Dynamic MRI assessment of signal and volume changes after treatment revealed that the tumor T2 signal was lower in tumors of rats treated with CA4P + pTACE than in those treated with pTACE alone. This result shows that following PTA, necrosis was the most significant, shrinkage was the greatest, and treatment was most effective in those treated with CA4P + pTACE. Postoperative laboratory tests and immunohistochemical analyses indicated that the Ki-67 anti-angiogenesis effect following CA4P treatment was greater in tumors of rats treated with CA4P + pTACE than in those treated with pTACE alone. The expression of CD31 and VEGF qualitatively was lower in those treated with CA4P + pTACE, and hepatorenal toxicity did not differ between the groups. These findings show that CA4P increases the synergistic effectiveness of TACE photo-thermal ablation treatment.

## Conclusions

Nanoparticles carrying Fe_2_O_3_-PDA-Dox as a new chemoembolization material allows for MRI-guided TACE-PTA, with increased nanoparticle uptake provided by the pre-injection of CA4P via the hepatic artery. This procedure increases the relative concentration of Fe_2_O_3_-PDA-Dox in tumor tissues, thereby increasing the anti-tumor effects of TACE-PTA.

## Supplementary information

**Additional file 1.** The magnetic effect, photothermal effect and PH trigger release effect of the Fe_2_O_3_-PDA-Dox nanoparticles.

## Data Availability

Data sharing not applicable to this article as no datasets were generated.

## Abbreviations

CA4P: Combretastatin A-4 phosphate disodium
Dox: Doxorubicin
HCC: Hepatocellular carcinoma
INR: International normalized ratio
PDA: Polydopamine
PTA: Photothermal ablation
TACE: Transarterial chemoembolization
