# Supplementary material for: Local application of doxorubicin- loaded Iron oxid nanoparticles and the vascular disrupting agent via the hepatic artery: chemoembolization–photothermal ablation treatment of hepatocellular carcinoma in rats
Source: Cancer Imaging. 2019 Nov 4;19:71. doi: 10.1186/s40644-019-0257-x (PMC6829940; doi:10.1186/s40644-019-0257-x)
Supplement: Supplementary file 1 — Additional file 1. The magnetic effect, photothermal effect and PH trigger release effect of the Fe2O3-PDA-Dox nanoparticles. [file 40644_2019_257_MOESM1_ESM.docx]

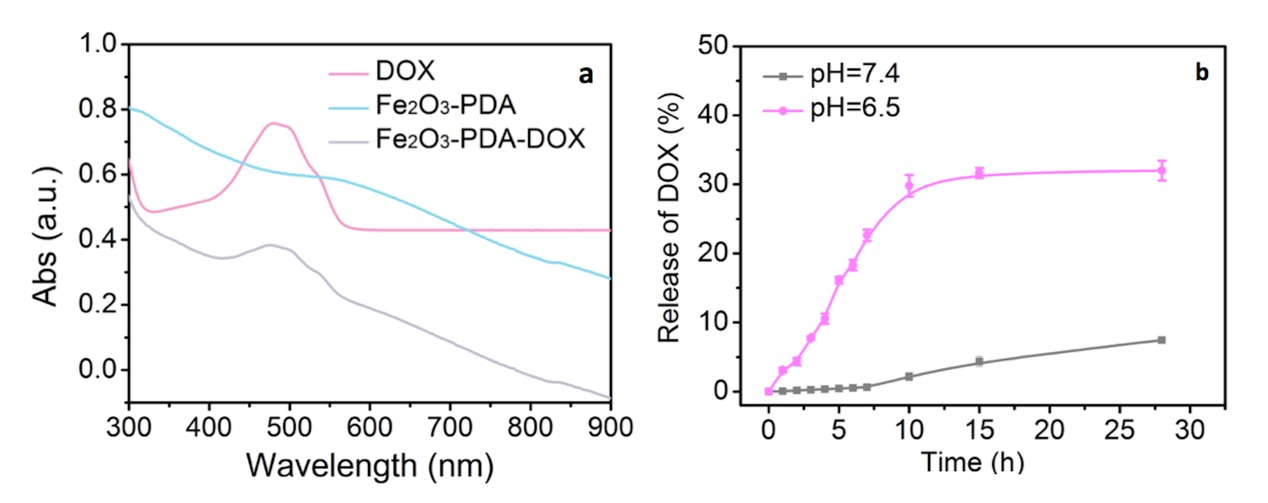


Figure S1. a. UV-VIS spectra of Dox, Fe_2_O_3_-PDA, Fe_2_O_3_-PDA-Dox; b. cumulative Dox drug release curves of Fe_2_O_3_-PDA-Dox nanoparticle solutions of differing pH.


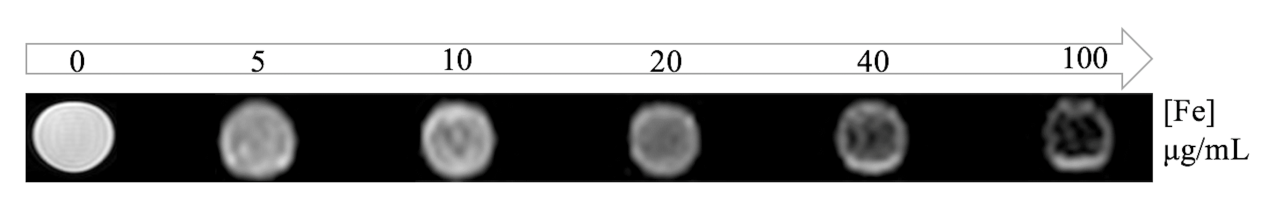


Figure S2. T2WI images of aqueous solutions with differing concentrations of Fe_2_O_3_-PDA-Dox nanoparticles.


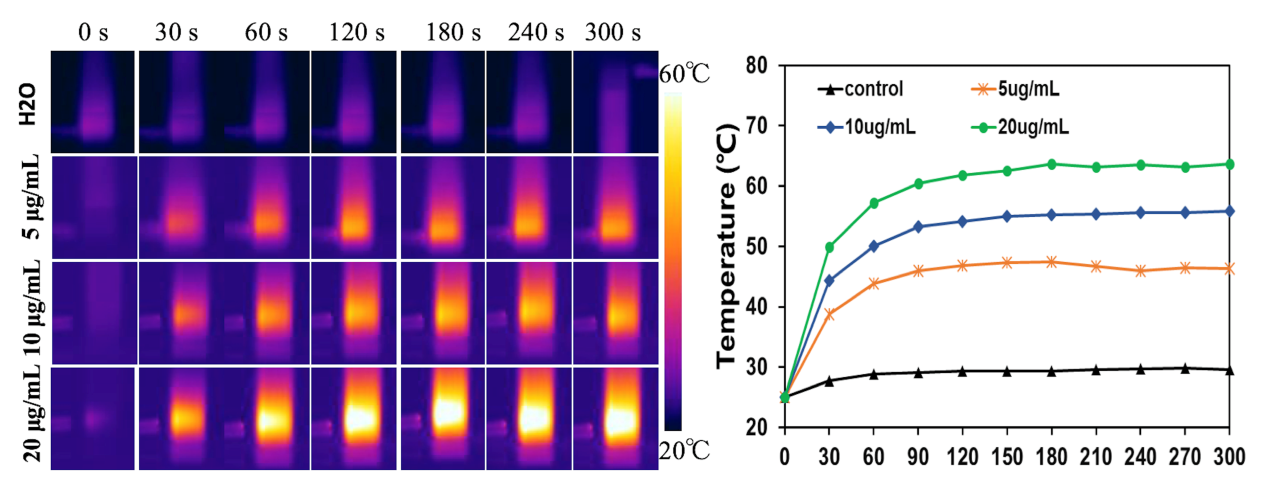


Figure S3. Photothermal response and heating curves of aqueous solutions with differing concentrations of Fe_2_O_3_-PDA-Dox nanoparticles.
